# CA-CWA: Channel-Aware Contention Window Adaption in IEEE 802.11ah for Soft Real-Time Industrial Applications

**DOI:** 10.3390/s19133002

**Published:** 2019-07-08

**Authors:** Yujun Cheng, Huachun Zhou, Dong Yang

**Affiliations:** School of Electronic and Information Engineering, Beijing Jiaotong University, Beijing 100044, China

**Keywords:** wireless local area network, IEEE 802.11ah, industrial IoT, medium access control, timeliness

## Abstract

In 2016, the IEEE task group ah (TGah) released a new standard called IEEE 802.11ah, and industrial Internet of Things (IoT) is one of its typical use cases. The restricted access window (RAW) is one of the core MAC mechanisms of IEEE 802.11ah, which aims to address the collision problem in the dense wireless networks. However, in each RAW period, stations still need to contend for the channel by Distributed Coordination Function and Enhanced Distributed Channel Access (DCF/EDCA), which cannot meet the real-time requirements of most industrial applications. In this paper, we propose a channel-aware contention window adaption (CA-CWA) algorithm. The algorithm dynamically adapts the contention window based on the channel status with an external interference discrimination ability, and improves the real-time performance of the IEEE 802.11ah. To validate the real-time performance of CA-CWA, we compared CA-CWA with two other backoff algorithms with an NS-3 simulator. The results illustrate that CA-CWA has better performance than the other two algorithms in terms of packet loss rate and average delay. Compared with the other two algorithms, CA-CWA is able to support industrial applications with higher deadline constraints under the same channel conditions in IEEE 802.11ah.

## 1. Introduction

In recent years, wireless communication has been widely adopted in the field of industrial communication systems [1,2]. Compared with traditional wired industrial communication systems [3] (e.g., Fieldbus and Industrial Ethernet), wireless communication does not require the deployment of expensive communication cables, and therefore they are cost-effective and easy to maintain. Thus, it is also very attractive for industrial soft real-time applications, such as soft real-time control systems [4] and multimedia embedded systems [5]. In soft real-time industrial systems, slight deadline misses are tolerable, as long as their impact is below some functional threshold, although this may affect quality of service and system accuracy to some extent. The tolerance degree depends on the different requirements of underlying industrial applications. Thus, when designing soft real-time systems, it is important to consider the deadline constraint and keep it below the threshold.

As one of the most widely deployed wireless technologies, IEEE 802.11 Wireless Local Area Network (WLAN) becomes a good candidate for various industrial wireless applications with different requirements [5,6]. However, WLAN was originally designed for high throughput applications. When it is adopted in the industrial context, a few issues are still to be resolved, such as energy efficiency, transmission range, interference and real-time performance. To provide a better support for IoT communications, the IEEE task group ah (TGah) released a new standard, called IEEE 802.11ah (marketed as Wi-Fi HaLow), and industrial automation is one of its typical use cases [7]. IEEE 802.11ah operates in the frequency band below 1 GHz, and supports up to 8192 nodes (sensors) in a WLAN with the transmission range up to 1 km. To address the collision problem for such a dense wireless network, the standard introduces a novel access mechanism called Restricted Access Window (RAW). The core idea of RAW is to limit the number of stations accessing the channel by a grouping-based medium access control (MAC) protocol. The stations are partitioned into groups, and the channel is split into slots to decrease collision probability in networks with thousands of stations.

Timeliness is usually dealt with at the MAC layer. In 802.11ah MAC, RAW mechanism can significantly reduce collisions and improve real-time performance. However, in each RAW slot, stations still need to contend for the channel by Distributed Coordination Function and Enhanced Distributed Channel Access (DCF/EDCA). While these MAC layer channel access schemes provide good real-time performance under light traffic, they have severe problems under congested network conditions when applied to real-time applications [8,9,10]. The original design intention of DCF/EDCA does not consider the deadline requirements, leading to unpredictable real-time performance of the industrial systems. Moreover, high external interference exists in the real industrial scenario, which brings high bit error rates in device communication [11]. The interference can seriously affect the network performance, which makes it harder to meet the real-time requirements of various industrial applications.

In this paper, the authors intend to improve the performance of the IEEE 802.11ah-based soft real-time networks in industrial scenario. The main contributions of this paper are summarized as follows:

- We propose a channel-aware contention window adaption (CA-CWA) algorithm, which dynamically increases and decreases the CW according to the channel status in order to improve the real-time performance of the RAW mechanism in IEEE 802.11ah.
- To eliminate the influence of the interference in real wireless environment, the CW adaption process is integrated with an external interference discrimination method. This method can improve the performance of CA-CWA algorithm effectively in the wireless environment with interference.

The remainder of the paper is organized as follows. Section 2 reviews the background and related work on this research. Section 3 presents the proposed CA-CWA algorithm in detail. Section 4 shows simulation results of the proposed algorithm in detail. Finally, conclusions are given in Section 5.

## 2. Background

### 2.1. IEEE 802.11 DCF and EDCA

**Figure 1:**
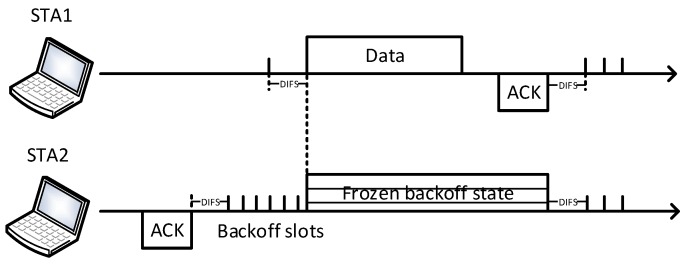
Example of the DCF mechanism.

As a fundamental MAC mechanism of the IEEE 802.11, the Distributed Coordination Function (DCF) is a simple and flexible scheme to share the medium among multiple stations. As shown in Figure 1, in DCF, stations contend for the chance of channel access by Carrier Sense Multiple Access mechanism with Collision Avoidance (CSMA/CA). When collisions happen, DCF adopts a Binary Exponential Backoff (BEB) algorithm to alleviate the congestion [12]. Two separate and distinct carrier-sensing functions are defined in IEEE 802.11 standard: Clear Channel Assessment (CCA) and the Network Allocation Vector (NAV). CCA is physical carrier sense, which determines whether the medium is idle or not, based on energy thresholds from the radio interface. NAV is virtual carrier sense, which is an indicator for the station to avoid potential conflicts by overhearing stations.

Before starting a new transmission, each station must sense the status of the channel. The station is permitted to initiate its transmission only if it finds the channel is idle in an additional random backoff period plus a DCF Interframe Space (DIFS) duration. Otherwise, the transmission must be frozen until the medium is idle again. The backoff duration is composed of a multiple of time slots. Each active station generates a uniformly random backoff value from [0, CW−1], where CW is the contention window size. The backoff value is the number of time slots that a station has to wait before transmission. In the first transmission, CW is set to minimal value $CW_{min}$, which is defined in the standard. When the transmission fails, the CW is doubled until it reaches maximum CW value $CW_{max}$. Once the CW reaches $CW_{max}$, the contention window is maintained at $CW_{max}$ even if the next transmissions are still unsuccessful. The CW is set back to $CW_{min}$ after a successful data transmission or when the retransmission counter exceeds the retry limit.

**Figure 2:**
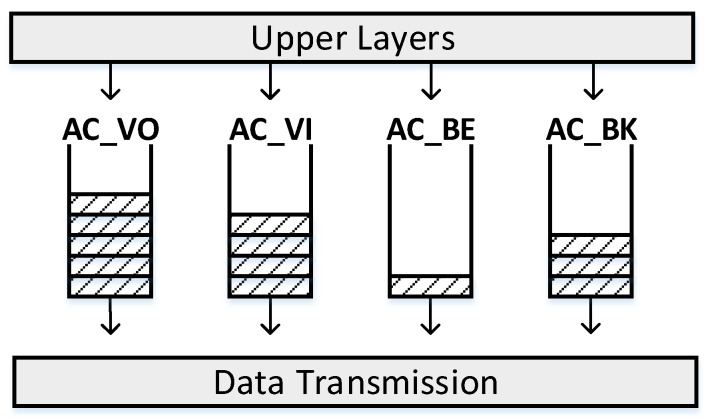
EDCA traffic priorities mapping.

To provide priority-based QoS for real-time applications, IEEE 802.11e task group enhances the DCF through a new channel access mechanism: Enhanced Distributed Channel Access (EDCA). As illustrated in Figure 2, four Access Categories (ACs) are defined in EDCA, namely voice (AC_VO), video (AC_VI), best-effort (AC_BE), and background (AC_BK) traffic. In EDCA, higher priority traffic uses shorter arbitration inter-frame space (AIFS). When an internal traffic collision happens, the higher priority access category obtains the data transmission chance, while the other ACs should restart the backoff procedures. With EDCA, high-priority traffic has a higher transmission chance than low-priority traffic by differentiating the backoff parameters for different ACs (Table 1).

### 2.2. IEEE 802.11ah RAW

Although IEEE 802.11ah inherits most of the basic IEEE 802.11 MAC features, several innovative MAC mechanisms are proposed to support the general requirements of the IoT applications. One of these novel MAC features is Restricted Access Window (RAW).

**Figure 3:**
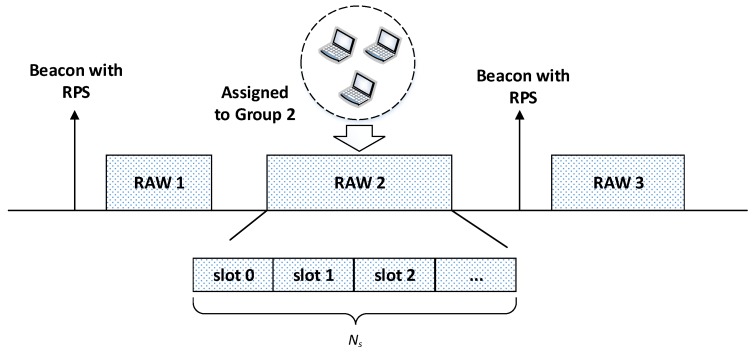
IEEE 802.11ah RAW structure.

The RAW mechanism aims to mitigate collisions in dense wireless networks, where a large number of stations are contending for channel access simultaneously. Specifically, the channel time is split into several intervals, namely the RAW periods and the shared channel airtime. As shown in Figure 3, only a portion of stations, namely the RAW stations, from a specific group are allowed to contend the channel in a particular RAW period. By contrast, all stations can compete for the channel in the shared channel airtime. The AP is responsible for assigning each RAW period to a group of stations by a beacon frame carrying a RAW Parameter Set (RPS), which is an information element that specifies the RAW related information, including the stations belonging to the group and the group start time. Besides, the RPS also contains the slot format, the number of RAW slots ($N_{s}$) and slot duration count sub-fields, which jointly determine the RAW slot duration as follows:

(1) D=500μs+C×120μs,

where *C* is slot duration count sub-field, and *D* is the RAW slot duration. The number of RAW slots $N_{s}$ and *C* are determined by the slot format sub-field. If the slot format sub-field is set to 1, each RAW period consists of at most eight RAW slots and the maximum value of *C* is 2047. Otherwise, each RAW period consists of at most 64 RAW slots and the maximum value of *C* is 255.

To make EDCA compatible with the RAW mechanism, each station adopts two backoff states of EDCA to manage data transmission inside and outside its assigned RAW slot, respectively [13]. The first backoff state is adopted outside RAW slots, in which all stations are permitted to compete for the channel. For the first backoff state, the station freezes its backoff timer at the start of each RAW period, and resumes the backoff timer at the end of the RAW period. The second backoff state is adopted inside RAW slots, where only the designated group of stations is permitted to contend for channel access. For the second backoff state, stations start backoff procedure at the start of their own RAW slot, and terminate their backoff procedure at the end of their RAW slot.

### 2.3. Contention Window Adaption

In IEEE 802.11 networks, it is extensively accepted that the backoff algorithm plays a significant role in achieving a high throughput and less medium access delay [14,15]. The IEEE 802.11 adopts a binary exponential backoff algorithm by default. As described in Section 2.1, when collisions happen, the BEB scheme simply exponentially doubles CW value to avoid repeated collisions, while it always resets the CW value to $CW_{min}$ after a successful transmission, assuming that the network is no longer congested. The fundamental problem is that BEB has no perception of the channel state, thus the algorithm does not know how to obtain an appropriate CW value to provide a better network performance.

Thus, considerable effort was devoted to improve the efficiency of the IEEE 802.11 backoff protocol. Several feedback-based schemes [14,15,16,17,18,19] have been proposed for adapting the station backoff to the present network conditions. In [16], an additional control is introduced on frame transmission for adaptation of CW according to the present network congestion. Further, the authors of [17] tuned CW by runtime estimation of the congestion condition and network status. In [14], the authors proposed a solution CCCW, which dynamically adjusts the CW size in both of saturated and unsaturated traffic conditions. The CW adaptation process in CCCW aims to achieve the optimal throughput. Recently, the authors of [15] proposed a delay-aware CW scheme adaption scheme called D2D, which tunes CW by the present delay level and channel congestion status of the network. However, these CW adaption schemes do not consider the influence of error-prone channel on the CW adjustment. On the other hand, several schemes [20,21] proposed the optimal configuration of the CW parameters in EDCA according to a predefined set of performance criteria. However, the theoretically derived optimal values rely on the actual measurement of network parameters such as number of contending stations, which is difficult to measure precisely in the real dynamic wireless environment.

## 3. The Proposed CA-CWA Algorithm

To fulfill the requirements of the industrial soft real-time applications, it is a good solution to improve the real-time performance of the IEEE 802.11ah networks by a better backoff algorithm. As discussed in Section 2.3, the most critical issue in designing backoff algorithm is to make it fully aware of the channel and network status. In industrial scenario, high external interference exists, which brings high bit error rates in device communication. The interference will significantly degrade the performance of the backoff algorithm in wireless channels [18,22]. Moreover, the designed CW adaption algorithm also needs to be carefully optimized for the two distinct backoff states of the RAW mechanism in IEEE 802.11ah, due to their different characteristics. To address these challenges, in this section, we first introduce a congestion status estimation scheme with interference discrimination ability by several observation measures. Then, a contention window adaption algorithm called CA-CWA is designed based on the congestion status estimation. Finally, the proposed CW adaption algorithm is integrated with the IEEE 802.11ah networks to provide better real-time performance.

### 3.1. Congestion Status Estimation

To provide a backoff algorithm in the 802.11 WLAN based on the congestion status, we need to first consider how to estimate the current network congestion level based on the available observation measures. In this work, we choose the parameter called channel busyness ratio (ρ) for capturing the channel status, which refers to the literature [14,19]. The channel busyness ratio is defined as the rate that a station finds the channel is busy during a certain time interval. Let $T_{i}$ be the slot length of the *i*th slot, in a given time interval which has *n* time slots, and the ratio ρ can be calculated as follows:

(2) $$\rho =\frac{\sum_{i=1}^{n}\alpha_{i}T_{i}}{\sum_{i=1}^{n}T_{i}}.$$

where $\alpha_{i}$ is the indicator function expressed as:

(3) $$\alpha_{i}=\left\{\begin{array}{c} 1,\;\mathrm{if}\;i\mathrm{th}\;\mathrm{slot}\;\mathrm{is}\;\mathrm{busy}\\ 0,\;\mathrm{if}\;i\mathrm{th}\;\mathrm{slot}\;\mathrm{is}\;\mathrm{idle} \end{array}\right..$$

In the IEEE 802.11 standard, every station has the ability of carrier sensing. Thus, $\alpha_{i}$ is easy to obtain without any additional hardware modification. However, in a real wireless scenario, both external interferences and transmission collisions can cause busy channels, which was not taken into account in the mentioned previous work. To obtain a more accurate estimation of $\alpha_{i}$, a method should be designed to discriminate between the external interferences and transmission collisions. Let *N* be the number of transmissions in a given period of time for an arbitrary station, and *S* of them are transmitted successfully (ACK is received). On the other hand, suppose there are *R* slots in which the station does not transmit, and *I* of them are idle, we can conduct the estimation of the collision probability $p_{c}$ and the channel error probability $p_{e}$ by maximum likelihood estimation method [23]:

(4) $$p_{c}=\frac{R-I}{R},$$

(5) $$p_{e}=1-\frac{1-(N-S)/N}{1-p_{c}}.$$

With the collision probability $p_{c}$ and the channel error probability $p_{e}$, the modified channel busyness ratio ($\rho^{\prime }$) can be calculated as follows:

(6) $$\rho^{\prime }=\frac{p_{c}}{p_{c}+p_{e}}\frac{\sum_{i=1}^{n}\alpha_{i}T_{i}}{\sum_{i=1}^{n}T_{i}}.$$

### 3.2. The Contention Window Adaption Scheme

In this section, we present the contention window adaption scheme based on the channel status estimation. In a general backoff scheme, a station should randomly select a backoff value from the interval [0, CW] before each transmission in order to avoid collision. Let $W_{k}$ be the CW size of the *k*th transmission attempt. The value of $W_{k}$ in BEB algorithm can be calculated as follows. If the CW limit CL ($CL=[log_{\omega }\frac{CW_{max}}{CW_{min}}]$) is greater than the retry limit RL, then,

(7) $$W_{k}=\omega^{k}CW_{min}.$$

otherwise,

(8) $$W_{k}=\left\{\begin{array}{c} \omega^{k}CW_{min},\;\mathrm{for}\;k\in \left[0,CL\right]\\ CW_{max},\;\mathrm{for}\;k\in \left[CL,RL\right] \end{array}\right.,$$

where $CW_{max}$, $CW_{min}$, and ω are the maximum CW size, the minimum CW size, and the backoff stage factor, respectively. In CA-CWA, only the CW update procedure is different from BEB after a success transmission and a failure transmission. In a fixed interval $T_{\rho }$, each station should observe the channel, and calculate the modified channel busyness ratio $\rho^{\prime }$ by Equation (6). Besides, to minimize the estimation bias introduced by burst traffic or interference, CA-CWA adopts an Exponentially Weighted Moving Average (EWMA) estimator to smoothen the estimated $\rho^{\prime }$. In an arbitrary interval $T_{\rho }^{j}$, the value of $\rho_{j}^{\prime }$ is updated according to the following rules:

(9) $${\rho_{avg}^{j}}^{\prime }=\left(1-\pi \right)\times \rho_{j}^{\prime }+\pi {\rho_{avg}^{j-1}}^{\prime },$$

where $\rho_{j}^{\prime }$ is the estimated $\rho^{\prime }$ in the interval $T_{\rho }^{j}$, ${\rho_{avg}^{j}}^{\prime }$ is the smoothed $\rho^{\prime }$ value for CW adaption, and π is the smoothing factor of the EWMA estimator, which determines the preserved number of historical values in the smoothing process. The ${\rho_{avg}}^{\prime }$ is updated continuously in each estimating interval $T_{\rho }$. The value of $T_{\rho }$ should be set appropriately to reflect the recent channel state better.

As mentioned in Section 2.1, EDCA defines four traffic types with different priorities. To ensure the priority mechanism still works properly, CA-CWA defines a decrement factor θ with different values for each type of traffic. Based on θ, the station is able to adjust its CW dynamically according to ${\rho_{avg}^{j}}^{\prime }$. The decrement factor of *n*th priority traffic ($\theta_{n}$) is defined as:

(10) $$\theta_{n}=\min\left\{\omega^{n}{\rho_{avg}^{j}}^{\prime },\theta_{max}\right\},\;n\in \left[1,4\right]$$

**Figure 4:**
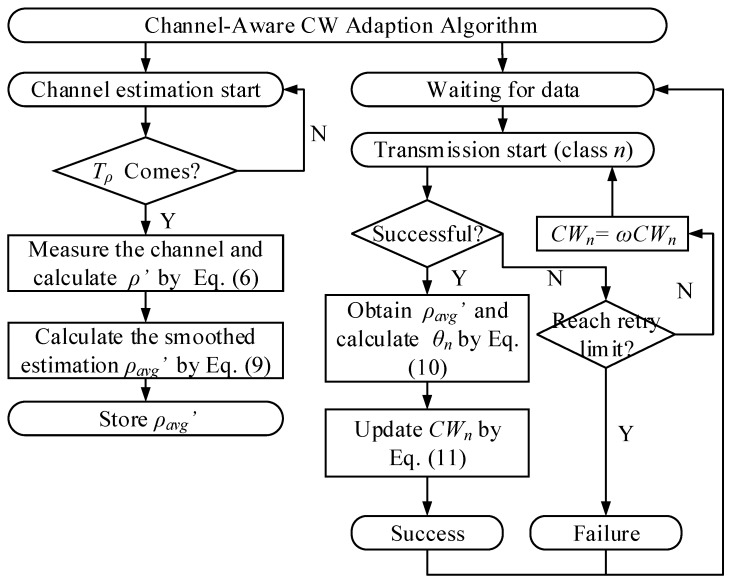
CA-CWA algorithm.

The traffic priority decreases gradually from *n* = 1 to *n* = 4, which ensures the higher priority class is able to adjust the CW parameter with a smaller θ. $\theta_{max}$ is a parameter that keeps the value of θ not be too large. An excessive value of θ might cause the reset CW value to be greater than the previous CW value. For each class *n*, $CW_{n}$ is updated after each successful transmission according to Equation (11):

(11) $$CW_{new}^{n}=\max\left\{CW_{min}^{n},\theta_{n}CW_{old}^{n}\right\},\;n\in \left[1,4\right]$$

where $CW_{old}^{n}$ is the CW value before an arbitrary successful transmission for class *n*, $CW_{new}^{n}$ is the updated CW value after the successful transmission. After each unsuccessful transmission, the CW of each class is doubled as long as the value of CW is less than $CW_{max}$, which is as same as the mechanism in BEB. Based on the channel status estimation, the aforementioned design of backoff adaption in CA-CWA follows a few principles which provide improved network performance in terms of timeliness. In a highly congested channel, a station should avoid blind resetting its CW to $CW_{min}$ after a successful transmission. In CA-CWA, a station will select an appropriate (relatively high) CW value according to the high value of θ in this situation. On the other hand, if the channel congestion reduces, a station will also reduce the value of θ and ensure a lower CW to minimize the access delay. In addition, to integrate CA-CWA into the IEEE 802.11ah protocol, CA-CWA defines a multiplier factor λ for the first backoff state (free contention period) in IEEE 802.11ah. For each station, the minimal CW is initialized to the product of the default $CW_{min}$ and λ because the channel is more likely to be congested. The CA-CWA algorithm is summarized in Algorithm 1, and a flowchart is illustrated in Figure 4 to make the algorithm process more clear. The two functions defined in Algorithm 1 are the two most upper arrows that appear in Figure 4. Besides, the specific values of the parameters in the algorithm will be introduced in the simulation part.

**Algorithm 1** Channel-aware CW adaption algorithm.
1:
**function** estmation
2:    **for** each fixed interval $T_{\rho }$ **do**3:        calculate $\rho^{\prime }$ by Equation (6)4:        ${\rho_{avg}}^{\prime }$← calculate the smoothed $\rho^{\prime }$ by Equation (9)5:        update ${\rho_{avg}}^{\prime }$6:    **end for**7:
**end function**
8: 9:
**function** CA-CWA
10:    /*initialization process*/11:    **if** second backoff state **then**12:        $CW_{min}$←$CW_{min}$13:    **else**14:        $CW_{min}$←$\lambda CW_{min}$15:    **end if**16:    initialize other parameters17:    18:    **while** (1) **do**19:        …20:        /*after a transmission triggered*/21:        **if** successful **then**22:           **for** n∈[1,4] **do**23:               obtain ${\rho_{avg}}^{\prime }$24:               calculate $\theta_{n}$ by Equation (10)25:               update $CW_{n}$ by Equation (11)26:               transmission end (successful)27:           **end for**28:        **else**29:           **if** reach retry limit **then**30:               transmission end (failed)31:           **else**32:               $CW_{n}$←ω$CW_{n}$33:           **end if**34:        **end if**35:    **end while**36:
**end function**



## 4. Performance Evaluation

In this section, we present our simulation results and analysis to demonstrate the real-time performance of the CA-CWA algorithm in IEEE 802.11ah networks.

### 4.1. Simulation Environment

**Figure 5:**
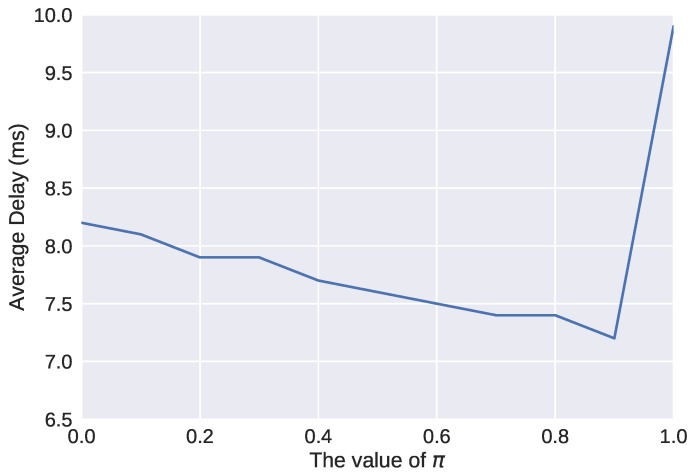
Smoothing factor effect on average delay.

The CA-CWA algorithm was implemented in the NS-3 simulator with IEEE 802.11ah modules, which is proposed in [24]. The simulation process was based on a general WLAN scenario, where one AP was located in the center, and other stations were randomly distributed around it within its communication range. Each station was installed with a UDP application that generated traffic with the interval of 0.05 s, and the packet size was set to 100 bytes due to the characteristics of small packet size in industrial scenario [25]. Only one transmission queue (AC_BE) was retained to focus on the competition among stations under the RAW mechanism, and $CW_{min}$ and $CW_{max}$ were set to the default values. The values of other network parameters were set by default according to Tian et al. [24]. On the other hand, the algorithm parameters were mainly determined by tests. For example, the smoothing factor π adopted in EWMA is usually recommended to be in the interval between 0.75 and 0.95 according to Lucas and Saccucci [26]. To obtain the optimal value of π, the relationship between the smoothing factor π and the network average delay was obtained through a test simulation. In the simulation, we adopted 10 non-RAW stations and 20 RAW stations, and the number of RAW slots $N_{s}$ was fixed to 1. The results are shown in Figure 5, and we finally set the value of π to 0.9 for the good performance. The main network parameters in the simulations are listed in Table 2.

### 4.2. Simulation Results

Because the proposed CA-CWA algorithm is mainly intended for industrial soft real-time applications, the simulation environment was adjusted to make it similar to the wireless conditions in industrial scenarios. According to the authors of [11,27], high interference exists in unstable and harsh industrial environments, which causes high bit error rates (BER = $10^{-2}$–$10^{-6}$) in industrial wireless communication. Thus, we adopted a loss model to introduce a packet loss ratio at approximately 2.5%. Besides, CA-CWA was compared with two other backoff algorithms, namely BEB and CCCW [14]. BEB is the default backoff algorithm in IEEE 802.11 protocol, and CCCW is another CW adaption algorithm based on the channel congestion status, which is described in Section 2.3.

In the simulation, we mainly focused on the real-time performance improvement that CA-CWA algorithm can bring for IEEE 802.11ah in industrial scenario. Thus, we chose average delay, packet loss ratio, and the delay distribution, which is the most important metric for the industrial soft real-time systems, to test the network performance. These metrics can reflect the real-time performance of the network very well. To eliminate random drift of the simulation results, each simulation was conducted five times, and the results are the average of the five.

To validate the characteristics of the algorithms in IEEE 802.11ah more precisely, the simulation was conducted in two simulation scenarios for the two backoff states.In the first simulation scenario, the number of non-RAW stations $N_{NRAW}$ (the stations which not support RAW) was fixed to 10. The number of RAW stations $N_{RAW}$ varied dynamically to obtain the real-time performance of the algorithms in the second backoff state (the backoff used in the RAW period). Besides, as one of the key parameters in the RAW mechanism, the number of RAW slots $N_{s}$ was also set to distinct values ($N_{s}$ = 1, 2 and 4) to show the influence of different RAW parameters on the network real-time performance. In fact, the influence of $N_{s}$ on network real-time performance has been discussed in the literature [28,29]. In simple terms, when $N_{s}$ increases and the other RAW parameters remain unchanged, the network delay will increase slightly under a lower load, but decrease slightly under a heavier load. The reason is elaborated in in detail below along with the simulation results.

**Figure 6:**
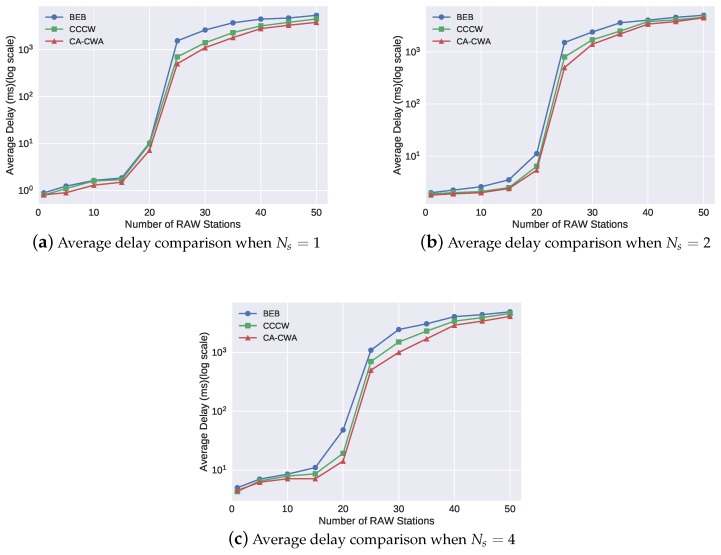
Performance of average delay of each algorithm.

**Figure 7:**
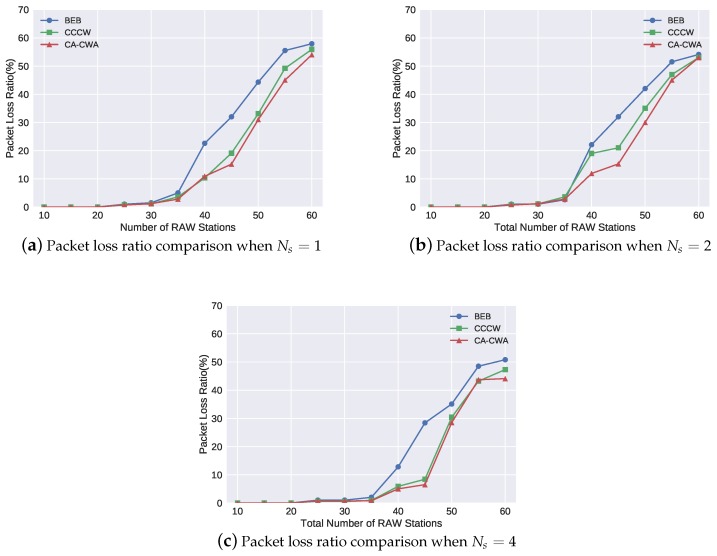
Performance of packet loss ratio of each algorithm.

Table 3 illustrates the change of the values of the core parameters in CA-CWA when varying the number of RAW stations, and $N_{s}$ was set to 1. To show the channel state estimation process of the CA-CWA in the simulation, we recorded the smoothed modified channel busyness ${\rho_{avg}^{j}}^{\prime }$. In each interval $T_{\rho }$, ${\rho_{avg}^{j}}^{\prime }$ was updated by Equation (9). Here, we use ${\rho_{avg}^{100}}^{\prime }$ to show the channel state because ${\rho_{avg}}^{\prime }$ tended to be stable after one hundred updates. Besides, the backoff decrement factor $\theta_{1}$, which was calculated by ${\rho_{avg}^{100}}^{\prime }$, is also listed in the table. We observed that the value of ${\rho_{avg}^{100}}^{\prime }$ and $\theta_{1}$ increased as $N_{RAW}$ increased, which is consistent with our intuition. The values of the ${\rho_{avg}^{100}}^{\prime }$ and $\theta_{1}$ were quite similar when $N_{s}$ was set to 2 and 4 in our simulation, and, therefore, the table only shows the change of the values when $N_{s}$ was set to 1. Moreover, it is easy to find the network real-time performance (average delay and packet loss ratio) under each value of ${\rho_{avg}^{100}}^{\prime }$ and $\theta_{1}$ by analyzing Figure 6 and Figure 7. For example, when $N_{RAW}$ equaled to 20, ${\rho_{avg}^{100}}^{\prime }$ and $\theta_{1}$ were 89% and 0.82, respectively, and the corresponding delay and packet loss ratio were 7.2 ms and 0, respectively.

Figure 6 and Figure 7 show the average delay and packet loss ratio of the different backoff algorithms under different RAW parameter settings. We observed that the objective values associated with each algorithm increased as the number of RAW stations increased. This is intuitively expected because the network is more congested if there are more stations transmitting their data. Comparing Figure 6a–c, we observed that the number of the RAW slots ($N_{s}$) can influence the delay performance slightly. For example, when the network was not congested ($N_{RAW}<15$), the average delay increased if the number of the RAW slots $N_{s}$ increased. When $N_{RAW}$ = 15, the average delay shown in Figure 6a–c was 1.2 ms, 2.24 ms and 7 ms, respectively. The reason is that the RAW mechanism only allows stations to transmit its data in its assigned slot. A station will wait for a longer time to transmit if there are more slots in a RAW. On the other hand, when the network is heavily loaded, adopting RAW can slightly alleviate network congestion, and obtain a lower average delay (although it is not obvious). The similar analysis result can be obtained for packet loss ratio when comparing Figure 7a–c.

Figure 6 and Figure 7 further show that the CA-CWA algorithm has better performance on average delay and packet loss ratio than the other two algorithms, regardless of the different number of RAW slots. We take the results in Figure 6a as an example; when $N_{RAW}$ was less than 15, all the three backoff algorithms performed well due to the non-congested channel condition. However, when $N_{RAW}$ was more than 20, the average delay of the network rose rapidly. In BEB, each station blindly reset its contention window to $CW_{min}$ after a successful transmission, which further aggravates the degree of channel congestion. Thus, BEB has the worst performance on average delay among the three algorithms. CCCW algorithm aims to optimize the network throughput by adaptive usage of contention window size. It significantly reduces collisions of the stations, and the average delay was up to 40% lower than the performance in BEB. However, CCCW does not consider the collisions caused by interference, which results in the adjusted CW value not matching the real channel condition. Thus, the average delay in CCCW was at most 30% higher comparing with the average delay in CA-CWA. On the other hand, Figure 7a shows the packet loss performance of the three backoff algorithms. The packet loss ratio of BEB increased dramatically from 5% to 23% when $N_{RAW}$ was 40. Compared with BEB, the packet loss ratio decreased up to 37.5% and 62.3% in CCCW and CA-CWA, respectively, when the network was congested ($N_{RAW}>=35$). A similar conclusion about average delay and packet loss ratio can also be drawn from the results in Figure 6b,c and Figure 7b,c.

**Figure 8:**
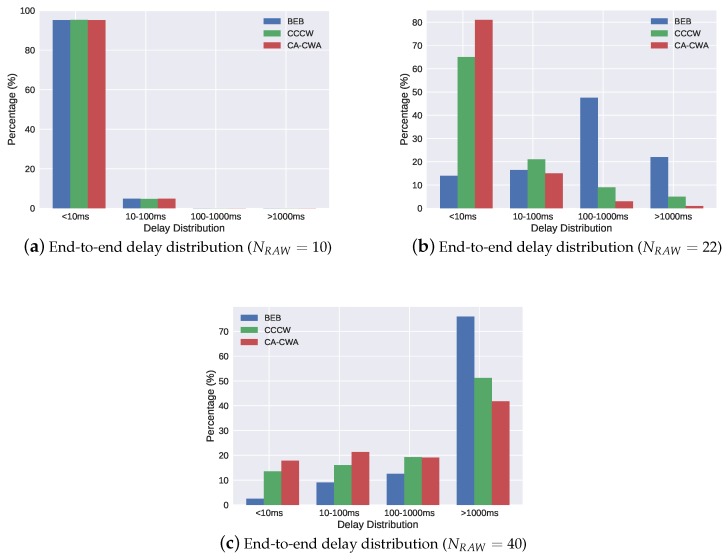
End-to-end delay distribution when $N_{s}=2$.

To indicate the real-time performance of the three algorithms more intuitively, we also plot histograms (Figure 8) to show the discrete distribution of the transmission delay in 10 RAW stations (Figure 8a), 22 RAW stations (Figure 8b) and 40 RAW stations (Figure 8c) scenarios, respectively. In Figure 8a, the end-to-end delay of 95% packets was below 10 ms in BEB, CCCW and CA-CWA, and only 5% packet delay was distributed between 10 and 100 ms. This is because the channel was not crowded in this scenario, and all the three backoff algorithms performed well. In the scenario shown in Figure 8b, the network was slightly congested. The end-to-end delay in BEB was mainly distributed between 100 and 1000 ms, which had the poorest performance among the three algorithms. By contrast, the end-to-end delay of CCCW and CA-CWA was mostly less than 10 ms due to their feature of CW adaption. Compared with BEB and CCCW, CA-CWA had 578% and 23.4% performance boost, respectively, when considering the percentage of delay distributed below 10 ms. In the scenario shown in Figure 8c, the end-to-end delay of the three algorithms was mainly distributed in the interval of more than 1000 ms, due to the heavily congested channel condition. However, compared with the other two algorithms, CA-CWA still had the largest proportion (17.8% for < 10 ms, and 21.3% for 10–100 ms) when considering the end-to-end delay distribution between 0 and 100 ms. In summary, CA-CWA is able to support industrial applications with higher deadline constraints under the same channel conditions in IEEE 802.11ah.

**Figure 9:**
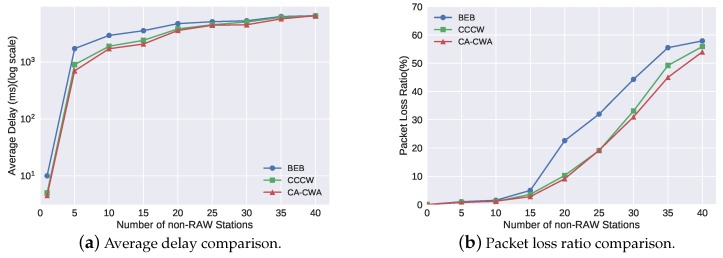
The real-time performance in the first backoff state.

The second simulation scenario was to verify the real-time performance of the first backoff state (the backoff used in the free contention period). Table 4 shows change of the values of the core parameters in CA-CWA when varying the number of non-RAW stations. In this scenario, the number of RAW stations $N_{RAW}$ was fixed to 20, and $N_{NRAW}$ varied from 0 to 40. As shown in Figure 9a,b, we observed that the average delay and packet loss ratio rose rapidly when $N_{NRAW}$ only equaled 5 and 20, respectively. This is because all stations could compete for channels in the first backoff state, which led to more congestion of the channel. The performance comparison of the three algorithms was similar to the discussion in the first scenario, which is disccussed in the previous paragraph. Figure 9a,b shows that the real-time performance of CA-CWA was superior to the other two algorithms too.

## 5. Conclusions

In this paper, we propose a channel aware contention window adaption (CA-CWA) algorithm for the real-time performance improvement of the IEEE 802.11ah-based industrial applications. The CA-CWA scheme adapts the CW according to a measurement based parameter called channel busyness ratio. Moreover, to eliminate the influence of the interference in real wireless environment on the algorithm, the channel busyness ratio is then modified with an interference discrimination method. To validate the performance of the proposed algorithm, we compared the real-time performance of CA-CWA with the other two algorithms, namely BEB and CCCW, in NS-3 simulator with IEEE 802.11ah modules. The results illustrate that CA-CWA has better performance than the other two algorithms in terms of packet loss rate and average delay. Moreover, CA-CWA has a lower delay distribution in the congested wireless condition. Thus, compared with the other two algorithms, CA-CWA is able to support industrial applications with higher deadline constraints under the same channel conditions in IEEE 802.11ah. As for the future research work, we plan to introduce a model considering both backoff schemes and RAW mechanism to provide a theoretical analysis of the network real-time performance, as well as to develop a new channel state estimation method based on several novel mechanisms, such as machine/deep learning techniques, to provide more accurate channel information. 

## Figures and Tables

**Table 1:** IEEE 802.11E EDCA parameter set.

AC	${\mathrm{CW}}_{\min}$	${\mathrm{CW}}_{\max}$	AIFSN	$T_{\mathrm{AIFS}}$
AC_VO	3	7	2	28 μs
AC_VI	7	15	2	28 μs
AC_BE	15	1023	3	37 μs
AC_BK	15	1023	7	73 μs

**Table 2:** Basic parameters in simulation.

Basic Parameters	
Reception energy threshold	−116.0 dbm
CCA threshold	−119.0 dbm
Noise figure	3 db
Channel bandwidth	1 MHz
Path loss model	Log-distance
Path loss exponent	3.67
Data rate	2.4 Mbps
Maximal distance between AP and stations	250 m
$CW_{min}$	15
$CW_{max}$	1023
UDP traffic interval	0.05 s
Packet payload size	100 bytes
**RAW Parameters**	
RAW slot format	0
*C*	100
*D*	12.5 ms
Number of group	1
**Algorithm Parameters**	
$T_{\rho }$	5 ms
λ	0.2
ω	2
π	0.9
$\theta_{max}$	0.82

**Table 3:** The calculated core algorithm value in the first scenario ($N_{s}$ = 1).

$N_{\mathrm{RAW}}$	1	5	10	15	20	25	30	35	40	45	50
${\rho_{avg}^{100}}^{\prime }$	2%	14%	35%	53%	89%	92%	94%	95%	98%	98%	98%
$\theta_{1}$	0.02	0.14	0.35	0.53	0.82	0.82	0.82	0.82	0.82	0.82	0.82

**Table 4:** The calculated core algorithm value in the second scenario.

$N_{\mathrm{RAW}}$	1	5	10	15	20	25	30
${\rho_{avg}^{100}}^{\prime }$	80%	92%	94%	95%	97%	98%	98%
$\theta_{1}$	0.80	0.82	0.82	0.82	0.82	0.82	0.82
